# Spatial and temporal inequalities in non-communicable disease mortality across the East African community: a Bayesian spatio-temporal analysis

**DOI:** 10.3389/fpubh.2026.1831129

**Published:** 2026-04-23

**Authors:** Tsikai Solomon Chinembiri, Onisimo Mutanga, Pedzisai Kowe

**Affiliations:** 1 Department of Environmental and Geographical Science, African Climate and Development Initiative (ACDI), University of Cape Town, Cape Town, South Africa; 2 College of Agricultural, Earth and Environmental Sciences, University of KwaZulu-Natal, Pietermaritzburg, South Africa; 3 Department of Geography, Environmental Sustainability and Resilience Building, Faculty of Social Sciences, Midlands State University, Gweru, Zimbabwe

**Keywords:** Bayesian spatio-temporal modeling, East Africa, environmental determinants, health inequalities, non-communicable diseases, shared-component modeling, spatial epidemiology

## Abstract

**Introduction:**

Non-communicable diseases (NCDs) are increasing rapidly across Sub-Saharan Africa, yet spatial inequalities in mortality across disease groups remain insufficiently characterized for effective regional health planning.

**Methods:**

We examined mortality from cardiovascular diseases, cancers, chronic respiratory diseases, and diabetes across seven East African Community countries from 2000 to 2019. Using WHO Global Health Observatory estimates, we constructed a balanced panel of 560 country-disease-year observations and fitted a multivariate Bayesian spatio-temporal shared-component model in INLA with environmental and socioeconomic covariates.

**Results:**

Socioeconomic context showed the strongest associations with mortality risk. GDP per capita was positively associated with NCD mortality, while healthcare expenditure, urbanization rate, and population density showed inverse associations. Environmental variables were weaker and statistically uncertain at country scale. Spatial patterns showed elevated cardiovascular and respiratory mortality risk in eastern areas, a west-to-east gradient for diabetes, and relatively uniform cancer mortality. Shared spatial effects identified persistent multi-disease high-risk clustering centered on Rwanda, Uganda, and Tanzania.

**Discussion:**

NCD mortality risk in the EAC is spatially structured and associated with contextual socioeconomic and environmental conditions, underscoring the importance of geographically targeted prevention strategies and spatially informed health-system planning.

## 1 Introduction

Non-communicable diseases (NCDs) have emerged as the dominant cause of global morbidity and mortality, accounting for over 70% of deaths annually (1). While historically considered diseases of affluence, NCDs are increasingly concentrated in low- and middle-income countries (LMICs), where demographic transitions, urbanization, and environmental exposures are rapidly altering health risk profiles (2). East Africa illustrates this shift in especially place-sensitive ways. Marked by rapid urban growth, persistent socio-economic inequities, and accelerating environmental change, the region faces a convergence of conditions that shape where and for whom health risks accumulate. In this context, the increasing burden of cardiovascular diseases, respiratory conditions, cancers, and diabetes mellitus threatens health-system resilience and jeopardizes sustainable development gains (3). Yet empirical work that explicitly maps the spatial and temporal patterning of NCD mortality, and links those patterns to modifiable contextual exposures, remains limited.

A growing literature documents relationships between NCD outcomes and environmental stressors including ambient air pollution, climatic variability, and urbanization (4, 5). PM_2.5_ exposure, for example, is consistently associated with elevated cardiovascular and respiratory mortality across diverse settings (6). Climate change has also altered precipitation and temperature regimes, with downstream consequences for food security, health services, and livelihoods that can indirectly influence NCD risks (7). Urbanization can expand access to care and infrastructure, but it may also intensify sedentary behavior, dietary risks, and exposure to harmful pollutants, producing uneven health effects across places (8).

Despite this evidence, many studies still treat environmental exposures and socioeconomic conditions as parallel rather than interacting processes. In particular, the interplay between structural socioeconomic factors and risks such as PM_2.5_ exposure, precipitation variability, and temperature extremes is often under-modeled, even though international evidence links these combined pressures to excess mortality (4, 5). As a result, relatively few analyses integrate multi-dimensional exposures into spatial-temporal frameworks that can quantify both shared and disease-specific NCD risks.

Bayesian hierarchical approaches have long been used for disease mapping and prediction in LMICs (9), but applications that jointly model multiple diseases while incorporating contextual covariates and geospatial structure remain uncommon, especially in East Africa. Moreover, many Bayesian disease-mapping studies rely on coarse or dated covariates and do not take advantage of newer developments in spatial priors (e.g., BYM2, PC priors) and hierarchical specifications that can improve interpretability and inferential clarity. This limits the ability to generate coordinated, policy-relevant evidence on NCD mortality risk that is comparable across borders and through time.

Macharia et al. (10) used Bayesian spatio-temporal models to show how environmental and health-system factors shape under-five mortality in Kenya. Xiong and Wang (11) applied Bayesian frameworks at the global scale to evaluate how economic development and air pollution jointly influence life expectancy. Similarly, Baer et al. (12) highlighted the value of multivariate disease mapping for capturing spatial dependence in health outcomes. Within East Africa, however, evidence has often remained national or subnational and focused on a single disease category. Spatial epidemiological studies of NCDs such as hypertension, diabetes, or respiratory diseases are increasing (13), but typically do not integrate multiple diseases within a unified modeling framework.

Although links between NCD risk and environmental or socio-economic factors are increasingly recognized, a key methodological gap persists: the shortage of unified, multi-disease, spatio-temporal models that account for co-occurring outcomes and their joint determinants. This gap is particularly consequential in East Africa, where limited surveillance infrastructure intersects with heterogeneity in climate, urban development, and public health investment. Because health disparities in the region are often spatially clustered, analytic approaches are needed that can distinguish shared vs. disease-specific spatial processes and track how these evolve over time. Without robust spatial evidence, policy-makers have fewer tools to target interventions, coordinate cross-sector strategies, or align environmental and health planning. While spatially resolved environmental and mortality data are increasingly available, their use remains constrained by methodological and capacity limitations.

Understanding the spatial and temporal dynamics of NCD mortality in East Africa therefore requires an integrated approach that captures both shared and disease-specific risk patterns. This study examines relative mortality risk for cardiovascular diseases, diabetes mellitus, malignant neoplasms, and respiratory diseases across the region from 2000 to 2019, while assessing how climate variability, air pollution, urbanization, and socioeconomic conditions jointly shape these outcomes. Implementing a multivariate Bayesian spatio-temporal model within the Integrated Nested Laplace Approximation (INLA) framework allows us to separate common and divergent contextual influences on NCD mortality risk across space and time.

Accordingly, this study is among the first to apply a shared-component Bayesian framework to NCD mortality across multiple disease groups in East Africa. By integrating harmonized environmental, climatic, and socioeconomic information from 2000–2019, it provides a spatially explicit account of chronic-disease mortality burden and its place-based patterning. Unlike approaches that analyse single NCD categories or isolated covariates, our framework estimates both disease-specific and shared spatial-temporal trends simultaneously. This supports stronger epidemiological inference and helps translate evidence into geographically targeted, context-aware policy responses. The analysis also identifies latent spatial risks that persist beyond observed determinants, underscoring the need for strengthened surveillance and integrated public health planning across the region.

The East African Community (EAC) region was selected because it represents one of the fastest-growing regions in Sub-Saharan Africa and is experiencing rapid epidemiological transition characterized by increasing non-communicable disease burdens alongside persistent infectious disease challenges. Despite these shifts, comparative spatial analyses of NCD mortality across the EAC remain limited. Examining the region collectively enables identification of cross-national patterns in mortality risk and provides insight into shared regional drivers of health inequality.

## 2 Methodology

### 2.1 Study area

**Figure 1:**
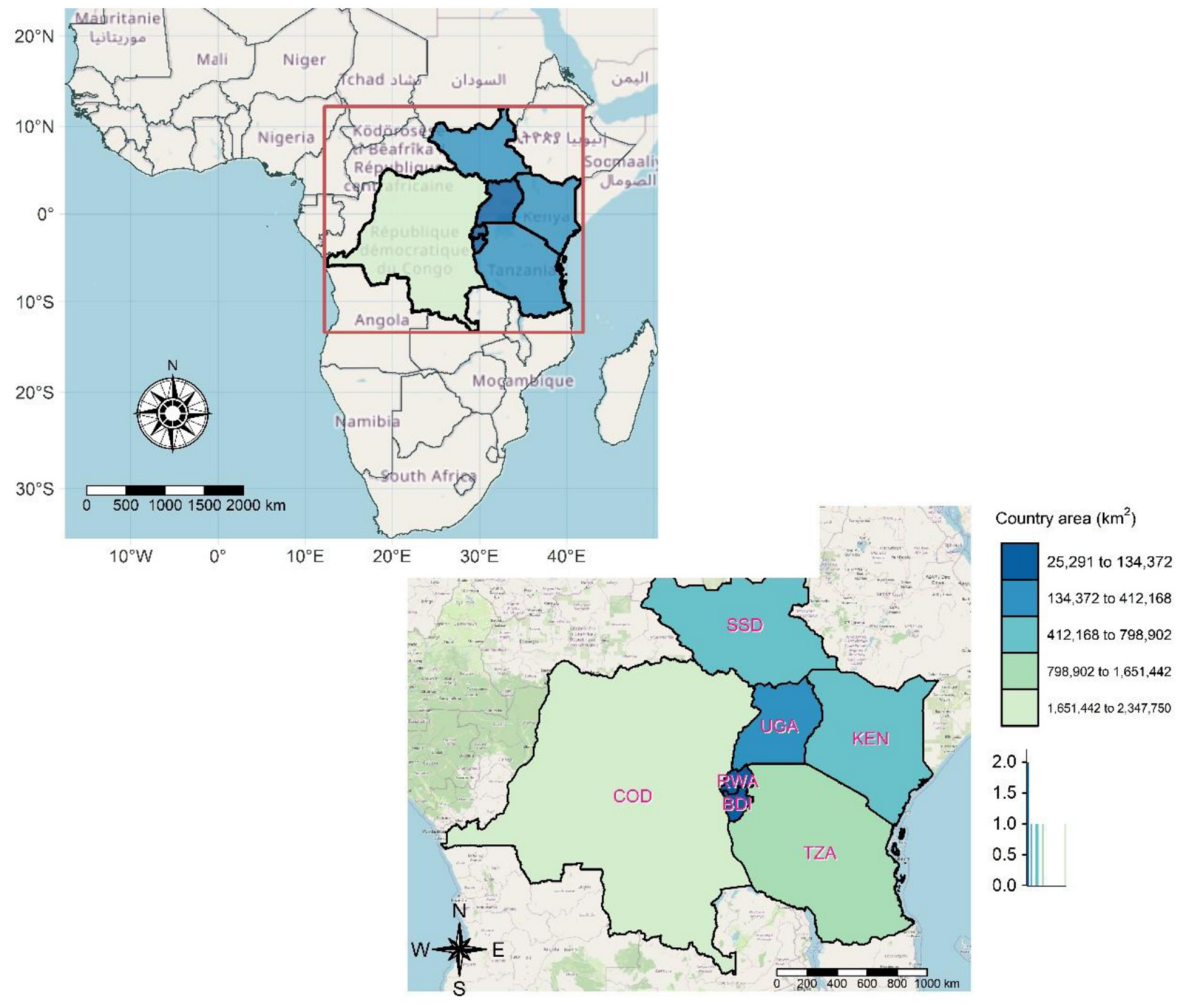
Spatial distribution of East African Community (EAC) countries within Sub-Saharan Africa, highlighting national boundaries and respective land areas (km^2^). BDI is Burundi, COD is the Democratic Republic of Congo, KEN is Kenya, RWA is Rwanda, TZA is Tanzania, UGA is Uganda and SSD is South Sudan.

This study focuses on countries that currently constitutes the East African Community (EAC) member states comprising of Burundi, the Democratic Republic of Congo (DRC), Kenya, Rwanda, South Sudan, Tanzania, and Uganda as in Figure 1 over a 20-year period from 2000 to 2019 (1). The rapid urban expansion, environmental vulnerability, and evolving demographic transitions collectively heighten the region's exposure to both modifiable and non-modifiable NCD risk factors. As one of Africa's fastest-growing regional blocs, the EAC faces increasing health system strain driven by economic disparity, climate variability, air pollution, and unplanned urbanization (2). Yet, spatial-temporal analyses of NCD mortality in this region remain sparse, making it a strategic and policy-relevant unit of analysis for understanding emerging health threats.

The study period (2000–2019) captures a critical transitional era marked by accelerated urban growth, climate change-induced environmental shifts, and a progressive shift in disease burden from infectious to chronic conditions. This 20-year window enables robust estimation of long-term relative mortality risk trends and spatial disparities across diverse ecological and socioeconomic settings within the EAC (3).

### 2.2 Multi-domain predictors of NCD mortality

We thematically grouped predictors into environmental and climatic drivers (e.g., PM_2.5_, NDVI, precipitation, and temperature) and socio-economic drivers (e.g., GDP per capita, healthcare expenditure, population density, and urbanization rate) to reflect distinct pathways through which contextual factors may influence NCD-related mortality risk across space and time.

#### 2.2.1 Environmental and climatic drivers

Vegetation dynamics were captured using NDVI from the Moderate Imaging Spectroradiometer (MODIS) MOD13A2 product, which offers 16-day composites at 500 m resolution and has supported global environmental monitoring since 2000 (4, 5). NDVI serves as a proxy for ecological stress and climate variability, both of which influence health outcomes (6, 7). Data were processed in Google Earth Engine (GEE) using zonal statistics to compute annual mean NDVI per country from 2000–2019 (8, 37). These indicators were integrated into the model to assess links between vegetation health and NCD mortality risk.

Air pollution data obtained as mean annual PM_2.5_ (PM_2.5_ exposure, measured in μg/m^3^) were extracted from the World Bank World Development Indicators (WDI) database via the WDI R package. PM_2.5_ exposure is a critical determinant of NCD burden, especially cardiovascular and respiratory conditions, with extensive epidemiological backing (4, 5). The inclusion of PM_2.5_ as a covariate is rooted in its well-established association with elevated risks of cardiovascular and respiratory mortality (9, 10). PM_2.5_ particles, due to their small size, can penetrate deep into the lungs and bloodstream, triggering inflammatory responses that exacerbate NCDs.

Long-term climatic exposure was assessed using mean annual temperature and mean annual precipitation, both of which are critical determinants of environmental health. Sustained high temperatures have been linked to increased physiological stress, particularly in vulnerable populations, and are known to elevate the risk of cardiovascular and other non-communicable diseases (11). Conversely, precipitation variability can influence food availability, water quality, and ecosystem stability, factors that indirectly shape the distribution and severity of NCD burdens (12). Climate data were sourced from WorldClim version 2.1, which provides globally consistent, high-resolution (30 arc-second) interpolated climate surfaces suitable for long-term spatial epidemiological analyses (13).

#### 2.2.2 Socio-economic drivers

Population density (people per square kilometer), also derived from the WDI database, serves as a surrogate for urban congestion and exposure risk (5). We selected population density as a proxy for urbanization, which influences exposure to environmental risks, healthcare access, and social determinants of health (5).

We also included GDP per capita, urbanization rate, and healthcare expenditure per capita to represent structural socio-economic determinants of health. GDP per capita reflects economic capacity, shaping both healthcare access and lifestyle risk factors such as diet and physical inactivity (14). Urbanization is linked to reduced physical activity, processed food consumption, and increased pollution which are key contributors to rising NCD prevalence (8). Healthcare expenditure per capita indicates national investment in health systems, with higher spending typically associated with better NCD prevention and management outcomes (15).

### 2.3 Modeling approach and estimation

We developed a joint Bayesian spatio-temporal model to estimate relative mortality risk for four major non-communicable disease (NCD) groups namely cardiovascular diseases, diabetes mellitus, malignant neoplasms and respiratory diseases across East Africa during 2000–2019. These disease groups were selected because they account for a substantial share of the regional NCD burden and, despite differences in proximate biological mechanisms, are shaped by overlapping population-level determinants, including air pollution, dietary transition, physical inactivity, urbanization, and health-system access. Within this framework, the shared component does not imply that the diseases are etiologically identical; rather, it captures latent spatial and temporal processes that may jointly influence multiple NCD outcomes at the population level. Disease-specific components then represent residual variation unique to each outcome after accounting for those common processes. This formulation is therefore epidemiologically appropriate for distinguishing cross-cutting contextual risk structures from disease-specific spatial and temporal heterogeneity (12, 16, 17).

The model was implemented using the Integrated Nested Laplace Approximation (INLA) framework in R, which enables efficient and accurate approximate Bayesian inference for latent Gaussian models without requiring Markov chain Monte Carlo (MCMC) sampling (16, 17, 33, 36).

### 2.4 Expected deaths and standardization

We obtained country-year NCD mortality estimates for 2000–2019 from the World Health Organization (WHO) Global Health Observatory (GHO), using the harmonized mortality series available at the time of data extraction (18, 19). These data provide country-level estimates of total NCD-related deaths and formed the basis for deriving expected mortality figures for cardiovascular diseases, cancers, chronic respiratory diseases, and diabetes across countries and years as in Equation 1:


$$\begin{array}{l} E_{itd}\;=\;\left(P_{it}\;\times \;R_{itd}\right)\;/100,000 \end{array}$$ (1)

where;

*E*_*itd*_ = the expected number of deaths for country *i*, year *t*, and disease group *d*.

*P*_*it*_ = the population of country *i* in year *t*.

*R*_*itd*_**=** age standardized mortality rate (per 100,000) for disease group *d* in country *i* and year *t*.

This standardization was necessary to account for differences in population age structure across countries and over time, thereby enabling more valid comparison of relative mortality risk across the study region (20).

### 2.5 Spatio-temporal disease modeling

Let *Y*_*itd*_ denote the observed number of deaths for country *i*, year *t*, and disease group *d*, and let *E*_*itd*_ denote the corresponding expected number of deaths derived from age-standardized mortality rates and population exposure. The expected deaths formulation is given in Equation 1, while the Poisson likelihood and structured additive predictor are given in Equations 2 and 3, respectively. We assumed:


$$\begin{array}{l} Y_{itd}\;\sim \;Poisson\left(E_{itd}\;\times \;\theta_{itd}\right) \end{array}$$ (2)

where θ_*itd*_ is the relative mortality risk. Under this parameterisation, θ_*itd*_ = 1 indicates that observed mortality equals the expected level, θ_*itd*_ > 1 indicates excess mortality relative to expectation, and θ_*itd*_ < 1 indicates lower-than-expected mortality. The logarithm of the relative mortality risk was then modeled as a structured additive predictor combining disease-specific intercepts, fixed effects, and latent spatial, temporal, and spatio-temporal random effects:


$$\begin{array}{l} \log\left(\theta_{itd}\right)=\alpha_{d}+x_{it}^{T}\beta +\lambda_{i}+u_{id}+\varphi_{t}+v_{td}+\psi_{itd} \end{array}$$ (3)

where:

$x_{it}^{T}\beta$ denotes the fixed effects of the observed environmental and socioeconomic covariates.

α_*d*_ is a disease-specific intercept.

λ_*i*_ denotes the shared spatial effect across diseases.

*u*_*id*_ denotes the disease-specific spatial effect.

φ_*t*_ denotes the shared temporal effect across diseases.

*v*_*td*_ denotes the disease-specific temporal effect.

ψ_*itd*_ denotes the disease-specific space-time interaction term.

In the INLA implementation, disease-specific intercepts were specified as independent and identically distributed random effects, shared and disease-specific spatial effects were modeled using the BYM2 specification, shared and disease-specific temporal effects were modeled using first-order random walks (RW1), and the spatio-temporal interaction was modeled as an *iid* effect grouped by disease (16, 17). This formulation enables the model to distinguish common contextual processes acting across diseases from residual disease-specific variation in space and time.

#### 2.5.1 Fixed effects and prior specification

All continuous predictors were standardized before model fitting to improve numerical stability and facilitate comparison of effect sizes across covariates. Accordingly, posterior fixed-effect coefficients are interpreted as the expected change in log-relative mortality risk associated with a one-standard-deviation increase in the corresponding predictor, conditional on the other variables in the model.

The model was fitted in R-INLA using the default prior specification. Under this specification, fixed effects follow the default Gaussian priors used by INLA, while the hyperparameters associated with the latent random effects follow the default prior settings of their respective model classes. For the BYM2 spatial effects, these defaults are based on the penalized-complexity framework, which regularizes model complexity by shrinking toward a simpler base specification while permitting data-driven departures where supported by the evidence (16, 17, 21).

Rather than specifying an explicit multivariate covariance structure across diseases, dependence between disease outcomes was represented through shared spatial and shared temporal latent components. This strategy preserves computational tractability within INLA while allowing common contextual processes and disease-specific deviations to be estimated simultaneously (16, 17).

Formal multicollinearity diagnostics were conducted using variance inflation factors (VIF), with a threshold of VIF < 5 adopted to indicate acceptable collinearity. None of the covariates exceeded this threshold, suggesting that problematic multicollinearity was not present among the predictors included in the model.

#### 2.5.2 Posterior diagnostics and model adequacy

The adequacy of the joint spatio-temporal model was evaluated using a set of Bayesian posterior diagnostics rather than relying solely on comparisons with alternative model specifications. Given the shared-component formulation and relatively small spatial domain, our emphasis was on checking predictive calibration, stability, and internal consistency rather than selecting among many competing models.

Specifically, model fit and predictive adequacy were examined using the Deviance Information Criterion (DIC) (22) and the Watanabe–Akaike Information Criterion (WAIC), which balance goodness-of-fit against model complexity. We also calculated the logarithm of the conditional predictive ordinate (LCPO) to assess out-of-sample predictive performance and inspected the number of CPO failures as a direct diagnostic of numerical stability in the leave-one-out predictive calculations.

Posterior predictive calibration was further evaluated using the Probability Integral Transform (PIT) values derived from the CPO diagnostics. Examination of the PIT distribution allowed assessment of whether the model produced systematically biased predictions, with an approximately uniform distribution indicating acceptable calibration. Observed-vs.-fitted comparisons were also inspected as a supplementary check on overall predictive agreement between the model and the mortality counts.

Together, these diagnostics provided complementary evidence of adequate model fit, predictive stability, and inferential reliability, supporting the appropriateness of the multivariate Bayesian shared-component model for characterizing spatio-temporal NCD mortality risk in the East African Community.

### 2.6 Spatial scale and aggregation considerations

The analysis was conducted at the national level because the WHO Global Health Observatory mortality estimates used in this study are harmonized and consistently available at country scale across the East African Community for the full study period. This spatial resolution enabled comparable cross-country analysis over time, but it also imposes important interpretive constraints. In particular, country-level aggregation may obscure substantial within-country heterogeneity in environmental exposures, health-system capacity, and socioeconomic conditions. This issue is especially relevant for geographically large and environmentally diverse countries such as the Democratic Republic of Congo and Tanzania, where national averages for PM_2.5_, NDVI, temperature, or precipitation may conceal marked subnational contrasts.

Accordingly, the spatial effects reported here should be interpreted as between-country differentials in relative mortality risk rather than as evidence of within-country spatial inequalities. The use of national averages also raises the possibility of attenuation of localized exposure-response relationships, particularly for environmental covariates that operate at finer spatial scales. In this sense, the weak or statistically uncertain environmental associations observed in the present study should not be interpreted as evidence that environmental determinants are unimportant, but rather as potentially reflecting scale mismatch between country-level mortality data and more spatially heterogeneous environmental processes. This limitation is consistent with the modifiable areal unit problem and with broader ecological constraints inherent in analyses based on aggregated spatial units (23–25).

## 3 Results

### 3.1 Multivariable determinants of cause-specific mortality risk

Because all continuous predictors were standardized prior to modeling, the coefficients in Table 1 represent associations per one-standard-deviation increase in each covariate.

**Table 1:** Posterior summaries of fixed-effect coefficients from the Bayesian spatio-temporal model estimating associations between environmental and socioeconomic predictors and relative mortality risk for major non-communicable diseases in the East African Community, 2000–2019.

Predictor	Mean	SD	2.5% Quantile	Median	97.5% Quantile	Mode	KLD
Mean annual NDVI	0.012	0.016	−0.019	0.012	0.043	0.012	0.0
Mean annual temperature	−0.008	0.078	−0.162	−0.008	0.147	−0.008	0.001
Mean annual precipitation	0.091	0.08	−0.066	0.091	0.248	0.091	0.001
GDP per capita	0.035	0.007	0.021	0.035	0.049	0.035	0.0
Health care expenditure	−0.022	0.006	−0.035	−0.022	−0.01	−0.022	0.0
Urbanization rate	−0.057	0.019	−0.095	−0.057	−0.02	−0.057	0.0
Population density	−0.058	0.008	−0.073	−0.058	−0.043	−0.058	0.0
PM_2.5_	0.004	0.011	−0.019	0.004	0.026	0.004	0.0

Values reported are the posterior mean, standard deviation (SD), 2.5% and 97.5% credible interval quantiles, median, mode, and Kullback–Leibler divergence (KLD).

From the results of Table 1, mean annual precipitation was positively associated with relative mortality risk (posterior mean = 0.091), but the 95% credible interval (−0.066 to 0.248) overlapped zero, indicating no strong evidence for a statistically significant effect. This suggests that, while precipitation may contribute to spatial variation in mortality risk, its influence remains uncertain in the presence of other covariates. Similarly, GDP per capita shows a consistently positive association (mean = 0.035), potentially reflecting greater detection and reporting of NCDs in wealthier regions, or shifting burden due to epidemiological transition.

In contrast, healthcare expenditure, urbanization rate, and population density exhibited negative associations with mortality risk. At the aggregate level, these patterns may reflect advantages associated with more urbanized and better-resourced settings, including closer physical access to health facilities, greater diagnostic availability, better referral systems, and earlier management of chronic disease. In this sense, the observed urban advantage may indicate that, within the present EAC context, the benefits of service concentration in urban centers currently outweigh some of the adverse behavioral or environmental risks associated with urban living. At the same time, this finding may also reflect relative neglect of rural populations, where delayed diagnosis, reduced continuity of care, and weaker mortality reporting systems may contribute both to poorer outcomes and to under-ascertainment of NCD deaths (19, 26).

PM_2.5_ concentration shows a small positive association (mean = 0.004), but with a wide credible interval overlapping zero, indicating uncertain influence at the aggregated scale. NDVI, a proxy for vegetation cover, also exhibits a small, positive but non-significant effect, while mean annual temperature has a near-zero mean effect with comparatively wider uncertainty. These weak environmental coefficients should not be interpreted as evidence of no environmental contribution to NCD mortality; rather, they likely reflect spatial aggregation, within-country heterogeneity, and mismatch between the scale at which environmental exposures operate and the scale at which mortality data were available.

### 3.2 Disease-specific spatial patterns of relative mortality risk (2000–2019)

**Figure 2:**
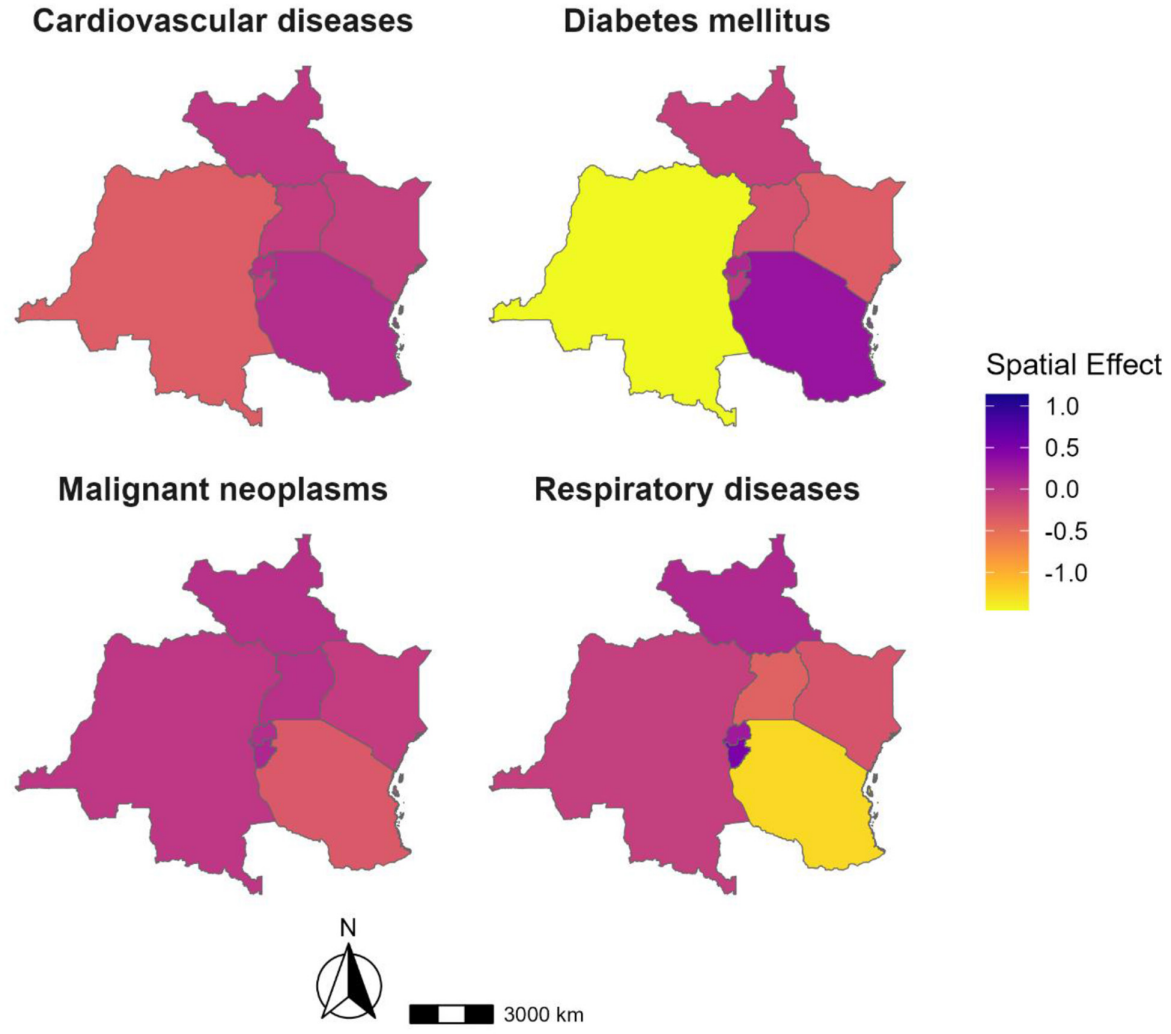
Disease-specific structured spatial effects for relative mortality risk in the East African Community, 2000–2019. Panels show posterior mean spatial effects from the joint Bayesian spatio-temporal model for cardiovascular diseases, diabetes mellitus, malignant neoplasms, and respiratory diseases. Positive values indicate areas with higher-than-average residual mortality risk after adjustment for observed covariates and temporal effects, whereas negative values indicate lower residual risk.

Cardiovascular diseases show elevated spatial effects in the eastern regions, potentially indicating localized risk factors such as unmeasured environmental exposures, healthcare access barriers, or behavioral patterns, while lower effects in the west suggest more favorable baseline conditions as illustrated in Figure 2. Again, as Figure 2 shows, diabetes mellitus presents a pronounced west-to-east gradient, with significantly lower residual risk in the western subregions and markedly higher effects in the southeast, consistent with urban clustering of risk factors not fully captured by modeled factors.

Malignant neoplasms exhibit relatively homogeneous spatial effects, suggesting uniformly limited access to early detection or treatment, though a modest central elevation may reflect local disparities in cancer care or environmental carcinogen exposure. Respiratory diseases show residual hotspots in central-eastern zones, aligning with expected geographies of poor indoor air quality and urban pollution, but also suggesting the influence of other latent exposures or structural determinants (Figure 2). Collectively, these residual spatial patterns underscore the importance of geographically targeted NCD control strategies, improved subnational health surveillance, and the need to further investigate unmeasured or inadequately captured spatial determinants of mortality risk in East Africa.

### 3.3 Shared spatial mortality pattern (2000–2019)

**Figure 3:**
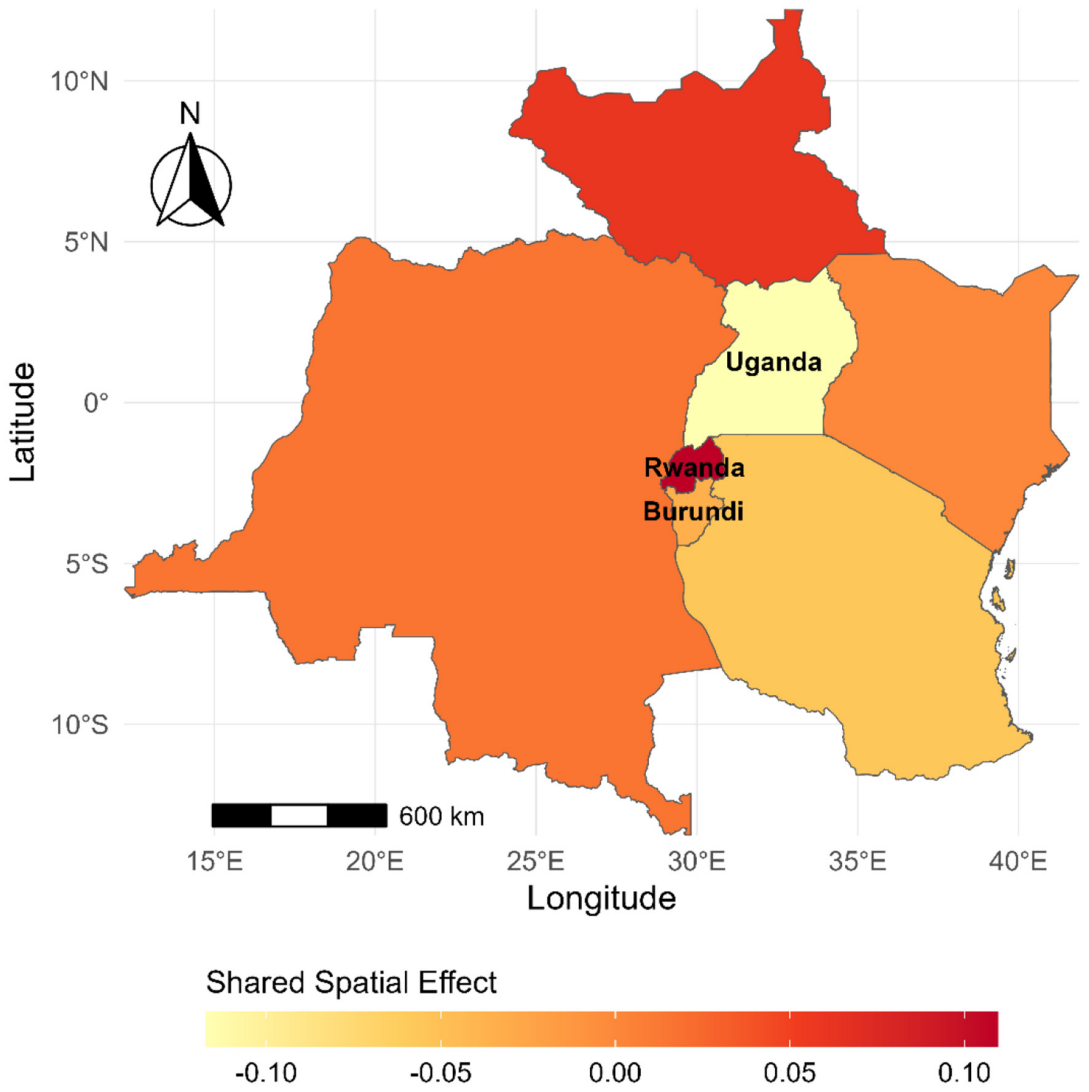
Shared structured spatial effect across the four major non-communicable disease groups in the East African Community, 2000–2019. The map shows the posterior mean shared spatial component from the joint Bayesian model. Positive values indicate geographic areas where excess mortality risk is common across disease groups after accounting for observed covariates, whereas negative values indicate comparatively lower shared residual risk.

Figure 3 illustrates regions where a consistent, unexplained excess (or deficit) in mortality risk persists across multiple disease categories.

Notably, areas in the north and around Rwanda exhibit strongly positive shared spatial effects, suggesting persistent elevated mortality risk not fully explained by the modeled NCD mortality determinants. These clusters may be indicative of unmeasured structural determinants such as health system limitations, conflict-related disruptions, regional health inequalities, or under-captured environmental exposures. In contrast, parts of eastern and southern regions show negative or near-zero shared spatial effects, indicating comparatively lower unexplained risk across diseases, possibly due to more robust infrastructure, better health access, or more complete explanatory power from the included covariates (Figure 3).

The shared pattern highlights the presence of underlying spatial processes jointly influencing multiple NCD outcomes, warranting integrative public health strategies. These findings support the development of regionally harmonized interventions and further investigation into cross-cutting spatial drivers of mortality risk, especially in high-burden zones.

### 3.4 Temporal and spatio-temporal mortality patterns (2000–2019)

**Figure 4:**
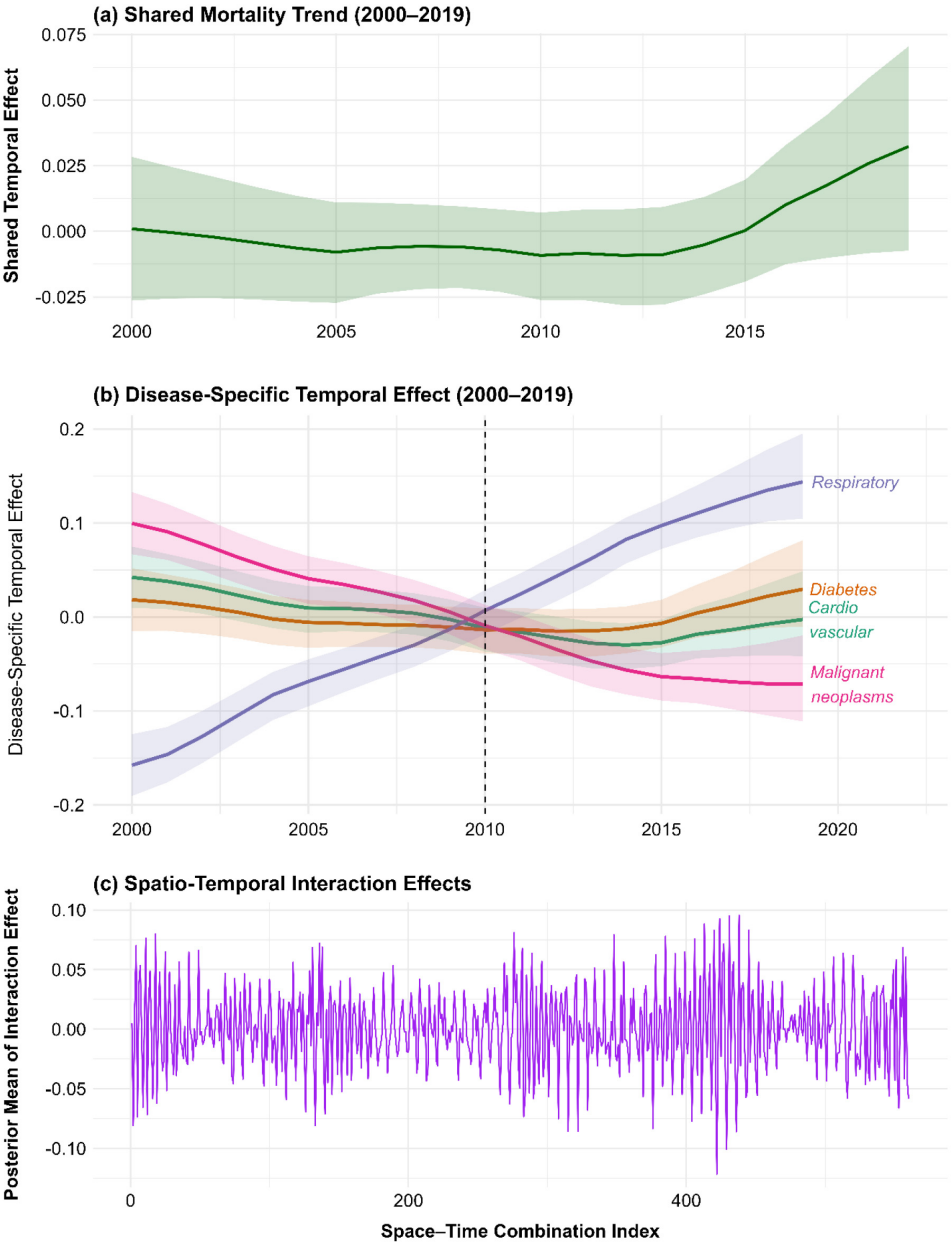
Temporal and spatio-temporal random effects for non-communicable disease mortality in the East African Community, 2000–2019. **(a)** shows the posterior mean shared temporal effect across all four disease groups. **(b)** shows disease-specific temporal deviations for cardiovascular diseases, diabetes mellitus, malignant neoplasms, and respiratory diseases. **(c)** shows posterior mean space-time interaction effects, representing localized departures from the main spatial and temporal trends.

Figure 4a shows the shared temporal trend, reflecting common mortality dynamics across all NCD categories. The overall effect is near-neutral in the early 2000s, followed by a slight decline through the mid-2000s, and a subsequent notable increase in shared mortality risk post-2015. This uptick may indicate regional-scale stressors such as worsening environmental exposures, health system strain, or epidemiological transitions affecting NCD outcomes collectively.

Figure 4b decomposes the disease-specific temporal effects, revealing distinct trends. Respiratory diseases exhibit a marked and continuous rise in temporal effect, indicating increasing mortality risk even after accounting for covariates, particularly after 2010. This trend may reflect growing exposure to air pollution or climate-sensitive respiratory risks. In contrast, malignant neoplasms show a declining temporal effect, possibly linked to improvements in detection or treatment infrastructure. Cardiovascular and diabetes-related mortality remain relatively stable, though diabetes exhibits a mild upward trend, hinting at a creeping epidemiological burden.

Figure 4c illustrates the posterior mean of the space–time interaction effects, capturing localized, time-specific deviations in mortality risk beyond what is explained by overall spatial and temporal trends. The fluctuating pattern with both positive and negative spikes suggests localized outbreaks or health system disruptions affecting mortality risk at specific times and locations, highlighting the necessity for dynamic, subnational health surveillance systems. Collectively, these temporal patterns underscore the evolving and heterogeneous nature of NCD mortality risk in East Africa. They emphasize the importance of anticipatory public health planning, disease-specific monitoring, and responsive policy to adapt to both broad and localized changes in risk profiles over time.

### 3.5 Spatio-temporal patterns of relative mortality risk (RR) in the EAC (2000–2019)

**Figure 5:**
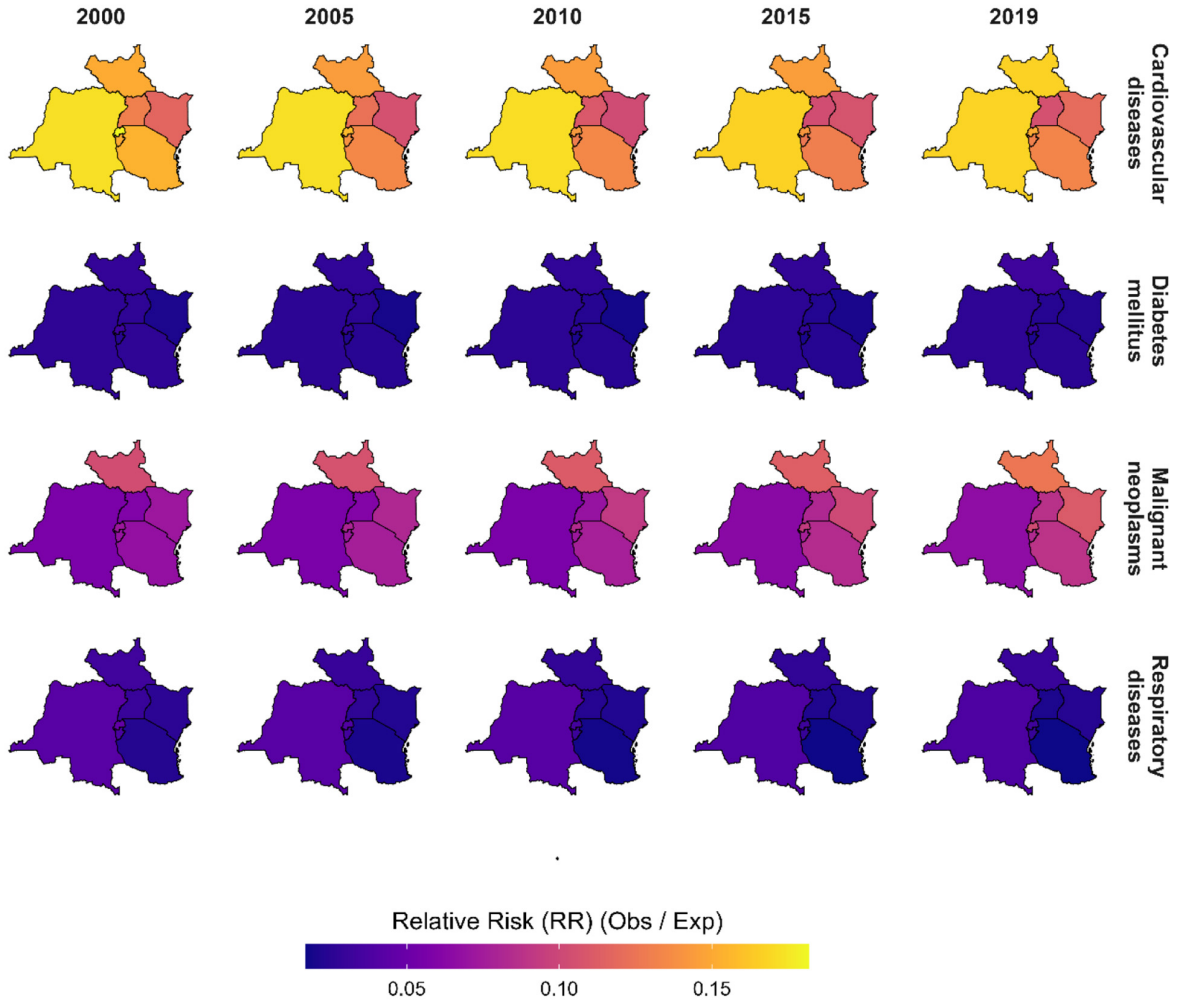
Spatio-temporal distribution of cause-specific relative mortality risk (RR) in the East African Community at selected time points from 2000 to 2019. Each panel maps the observed-to-expected mortality ratio for cardiovascular diseases, diabetes mellitus, malignant neoplasms, and respiratory diseases. Values greater than 1 indicate excess mortality relative to the standardized expectation, whereas values below 1 indicate lower-than-expected mortality.

Cardiovascular mortality consistently displays elevated relative risk in the north-eastern and eastern subregions across all five-yearly time points, with modest temporal fluctuations (Figure 5). This persistent spatial clustering suggests enduring structural determinants such as urban lifestyle risk factors or healthcare access disparities underlying the observed excess risk. The relative risk for diabetes mellitus remains uniformly low across the region throughout the study period, with minimal spatial differentiation. This pattern suggests a widespread but low-attributable mortality burden, potentially reflecting under-diagnosis, low healthcare utilization, or delayed mortality reporting for diabetes-related deaths.

Malignant neoplasms show a consistent concentration of higher relative risk in central and northern areas, with spatial persistence over time (Figure 5). This may reflect geographic disparities in cancer incidence, exposure to environmental carcinogens, or inequitable access to early detection and oncology services. Respiratory disease-related mortality appears more spatially diffuse, with relative risk remaining low across most regions and years, though some eastern and central pockets indicate slight elevations. This suggests a generally low mortality burden with localized variations, likely tied to specific environmental or demographic exposures not fully captured by model covariates. The observed patterns underscore the importance of temporally and geographically targeted strategies for NCD control. Again, as illustrated in Figure 5, the spatial persistence of high relative risk areas, particularly for cardiovascular and neoplastic diseases, calls for subnational health system strengthening, localized preventive interventions, and improved access to diagnostics and treatment across the EAC.

### 3.6 Posterior diagnostics and model adequacy

#### 3.6.1 Unexplained spatiotemporal patterns of NCD mortality risk in EAC countries

**Figure 6:**
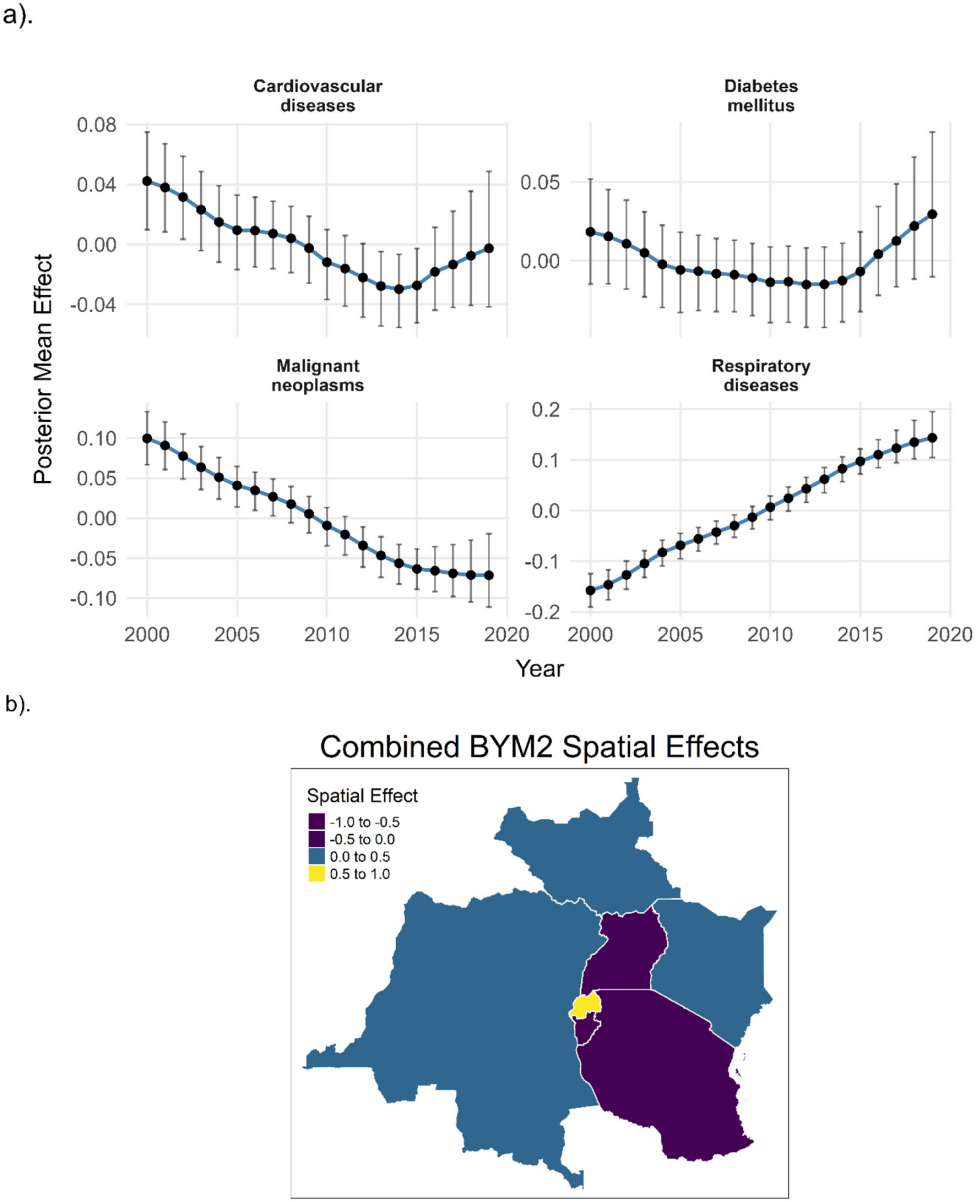
Posterior residual temporal and spatial random effects for non-communicable disease mortality across East African Community countries, 2000–2019. **(a)** shows disease-specific residual temporal effects from the Bayesian random-walk specification after adjustment for observed covariates, shared structure, and expected deaths; vertical bars denote 95% credible intervals. **(b)** maps the posterior mean combined residual spatial effects from the BYM2 specification, highlighting areas where unexplained mortality risk remains elevated or reduced after model adjustment.

Figure 6a illustrates diagnostic insights from the model's temporal effects. Malignant neoplasms and cardiovascular diseases exhibit a declining residual temporal trend from 2000 to around 2015, potentially reflecting improvements in healthcare or the model's increasing ability to account for structured drivers. In contrast, diabetes mellitus and respiratory diseases show a rising unexplained temporal effect, particularly after 2010, suggesting a growing influence of unmeasured factors such as lifestyle transitions or pollution dynamics. The widening uncertainty intervals for diabetes and respiratory outcomes in later years may indicate increased variability or data sparsity.

Figure 6b maps the combined spatial effects from the BYM2 model, representing posterior estimates of location-specific residual risk averaged across all NCD outcomes. The purple shading over parts of Uganda and Rwanda indicates persistent, elevated unexplained mortality risk, despite adjustment for known determinants. Conversely, most of the western and coastal subregions exhibit near-zero or negative spatial effects, implying either sufficient explanatory power from covariates or genuinely lower underlying risk.

The clustering of high residual risk in central zones may point to structural health inequities, diagnostic capacity gaps, or localized environmental or socio-political determinants not explicitly included in the model. These diagnostics support the robustness of the joint modeling framework while revealing residual spatiotemporal risk requiring further investigation. Findings highlight the need for improved surveillance and modeling of unmeasured or latent factors. Future models should integrate context-specific exposures and finer-resolution data to better guide targeted interventions.

Model diagnostics indicated satisfactory model performance. The fitted model produced a Deviance Information Criterion (DIC) of 7,072.83 and a Watanabe–Akaike Information Criterion (WAIC) of 6,939.86. The logarithmic Conditional Predictive Ordinate (LCPO) was −3,870.31, indicating reasonable predictive performance. No CPO failures were detected, suggesting stable estimation of leave-one-out predictive densities. Posterior predictive checks based on Probability Integral Transform (PIT) values showed no systematic deviations from uniformity, indicating adequate predictive calibration.

## 4 Discussion

This study provides new insights into the joint spatio-temporal distribution of non-communicable disease (NCD) mortality in East Africa, with a focus on four major causes: cardiovascular diseases, diabetes mellitus, malignant neoplasms, and respiratory diseases. Because the analysis is ecological and based on country-level panel data, the associations reported should be interpreted as contextual rather than causal, and they do not necessarily reflect individual-level relationships.

The multivariable analysis highlights the nuanced role of structural and environmental factors in shaping non-communicable disease (NCD) mortality patterns across the region. While GDP per capita showed a small but consistently positive association with NCD mortality, this relationship should not be interpreted causally. One plausible explanation is epidemiological transition, whereby economic development is accompanied by dietary change, reduced physical activity, and increased exposure to behavioral risk factors associated with chronic disease. However, the association may also reflect surveillance and ascertainment effects (38). Countries with higher income levels often have stronger diagnostic infrastructure, more complete civil registration and vital statistics systems, and better cause-of-death attribution, all of which increase the likelihood that NCD deaths are detected and recorded. Conversely, lower recorded mortality in poorer settings may partly reflect under-diagnosis or under-reporting rather than lower underlying burden. The GDP coefficient should therefore be interpreted as capturing a combination of structural development, epidemiological transition, and differential mortality ascertainment (19, 27).

In contrast, healthcare expenditure, urbanization rate, and population density exhibited negative associations with mortality risk. At the aggregate level, these patterns may reflect advantages associated with more urbanized and better-resourced settings, including closer physical access to health facilities, greater diagnostic availability, better referral systems, and earlier management of chronic disease. In this sense, the observed urban advantage may indicate that, within the present EAC context, the benefits of service concentration in urban centers currently outweigh some of the adverse behavioral or environmental risks associated with urban living. At the same time, this finding may also reflect relative neglect of rural populations, where delayed diagnosis, reduced continuity of care, and weaker mortality reporting systems may contribute both to poorer outcomes and to under-ascertainment of NCD deaths. The inverse association should therefore be interpreted cautiously as a contextual ecological pattern rather than evidence that urbanization is intrinsically protective (19, 26).

The environmental covariates included in the model including PM_2.5_ concentrations, NDVI, temperature, and precipitation showed weak or statistically uncertain associations with NCD mortality at the country level. While environmental exposures are widely recognized as important determinants of chronic disease, their effects are often spatially heterogeneous and may not be fully detectable when both mortality and exposure data are aggregated to national means. Accordingly, the absence of strong environmental coefficients in the present analysis should not be interpreted as evidence that environmental determinants are unimportant; rather, it may reflect scale mismatch, attenuation due to national averaging, and unobserved subnational exposure contrasts (41).

Future research using subnational or city-level datasets would provide a more appropriate framework for evaluating environmental determinants of NCD mortality and may yield stronger empirical evidence regarding the role of environmental exposures in shaping spatial health inequalities across the region.

Furthermore, environmental risk factors for NCDs often operate at finer spatial and temporal scales than those captured in national datasets. For example, exposure to air pollution or urban heat islands tends to vary significantly within cities and across ecological zones, while vegetation-related health benefits associated with NDVI may depend on local accessibility to green space rather than national averages (34, 35, 43, 44). Consequently, the country-level resolution used in this study may obscure important local environmental-health relationships. Future research using subnational or city-level datasets would therefore provide a more appropriate framework for evaluating environmental determinants of NCD mortality and could yield stronger empirical evidence regarding the role of environmental exposures in shaping spatial health inequalities across the region.

Similar trends have been observed in other regional studies where urban centers offer greater diagnostic and treatment availability (28). However, residual spatial patterns suggest that these effects do not uniformly protect against mortality, highlighting the role of unmeasured determinants or system-level inequities.

The shared spatial effect map reveals persistently elevated mortality risk in parts of Tanzania, Uganda, and Rwanda. These patterns remain after adjusting for modeled covariates and are suggestive of broader structural determinants such as regional health system gaps, inequitable access to care, or under-captured exposures like conflict disruption or indoor air pollution. These findings resonate with similar spatial clustering of health inequities observed in West Africa (29) and underscore the need for geographically targeted intervention strategies.

Disease-specific spatial effects further illuminate the differential epidemiology of the four NCDs. Cardiovascular mortality consistently concentrated in the east, potentially reflects environmental exposures, dietary patterns, or stress-related urbanization gradients. This corroborates earlier work noting elevated hypertension and stroke prevalence in urban East Africa (30). In contrast, diabetes mortality showed a distinctive west-to-east risk gradient, with particularly high risk in southeastern areas (31, 42). This may signal an emerging metabolic risk burden in transitioning peri-urban zones, consistent with trends in obesogenic environments and food system shifts reported elsewhere in sub-Saharan Africa (31).

The spatial pattern for malignant neoplasms was relatively homogeneous, implying region-wide limitations in early detection and treatment services, rather than geographically differentiated risk exposures. Prior studies across SSA have reported delayed presentation and inadequate oncology services as key drivers of cancer mortality (16). The modest central elevation in risk could reflect local environmental exposures (e.g., mining-related carcinogens) or healthcare access gaps (26, 39, 40). Respiratory diseases displayed scattered risk hotspots in central and eastern zones, aligning with biomass fuel usage and urban air pollution gradients, echoing findings from recent WHO urban health assessments.

Temporal decomposition of the shared mortality trend revealed an uptick after 2015, suggesting broad regional drivers, potentially linked to growing environmental stressors, health system strain, or changing behavioral risk profiles. Disease-specific temporal trends painted a more nuanced picture: respiratory diseases showed a steadily increasing trajectory, in line with worsening air quality, while malignant neoplasms trended downward, perhaps reflecting gradual improvements in detection or palliative infrastructure. The relatively stable cardiovascular and diabetes trends might indicate both competing risks and a masking effect due to broader structural dynamics.

Residual temporal effects also provided diagnostic value. The observed decline in unexplained temporal risk for cardiovascular diseases and malignancies after 2010 suggests improving model fit or capture of structured drivers, possibly reflecting expanded health investments or awareness. Conversely, the post-2010 rise in unexplained temporal effects for diabetes and respiratory diseases implies emerging determinants that remain unaccounted for. This highlights the growing need to integrate time-sensitive behavioral, environmental, and health system indicators into future modeling frameworks (18).

Model validation results confirm that the joint spatio-temporal framework adequately captured both structured and residual variation in mortality risk. The reported DIC (7072.72), WAIC (6939.92), and LCPO (−3870.44) all indicate strong internal model fit. While no alternative models were directly compared given the model's theoretical specification and shared structure, diagnostics from posterior spatial and temporal effects confirm coherent epidemiological signals and support the use of shared components to capture cross-disease dependencies (32). This approach offers interpretative efficiency and epidemiological robustness, as also supported in prior multivariate Bayesian frameworks for disease mapping (20).

A key limitation of this study relates to the spatial scale of the analysis. Because mortality data from the WHO Global Health Observatory are harmonized at the national level, the models were estimated using countries as the primary spatial unit. This introduces the potential for the modifiable areal unit problem, whereby statistical relationships may vary depending on the spatial scale or zoning of aggregation (23–25, 40). As a result, the findings primarily capture between-country differences rather than within-country inequalities. Future research using province-, district-, or city-level mortality data would provide a more detailed understanding of spatial health disparities, although such harmonized subnational datasets are not yet consistently available across the region.

A second limitation concerns the mortality input data themselves. The WHO Global Health Observatory provides invaluable harmonized estimates for comparative analysis, but these estimates remain partly dependent on the completeness and quality of the underlying national civil registration, cause-of-death certification, and health information systems. Across the East African Community, these systems vary in coverage, diagnostic capacity, and reporting completeness, and such differences may be especially pronounced in settings affected by conflict, weak mortality surveillance, or prolonged resource constraints. Consequently, some of the observed between-country variation in recorded NCD mortality may reflect differences in ascertainment and reporting practice in addition to genuine epidemiological differences (18, 19).

In addition, the study design is ecological in nature. The estimated associations operate at the country-year level and should not be interpreted as individual-level relationships. As with other aggregate spatial analyses, the findings are susceptible to ecological fallacy, whereby associations observed across countries may not hold for individuals or smaller geographic units. Residual confounding is also possible because several relevant determinants of NCD mortality, including behavioral risk factors, obesity, tobacco exposure, household air pollution, healthcare quality, and diagnostic access, were not available in harmonized form for all countries and years (23, 24).

### 4.1 Policy implications for regional health planning

The identification of persistent multi-disease high-risk clusters across parts of Rwanda, Uganda, and Tanzania highlights the need for geographically targeted prevention and intervention strategies. Rather than relying solely on nationally uniform policies, regional health planning initiatives within the East African Community could prioritize spatially informed interventions in areas where multiple non-communicable disease (NCD) risks converge. Such interventions may include integrated NCD screening programs, strengthened primary healthcare services, and targeted public health campaigns focusing on lifestyle-related risk factors.

In addition, the integration of environmental monitoring, particularly air-quality surveillance and urban environmental management may support early identification of emerging health risks in rapidly urbanizing regions. Spatial modeling approaches such as those presented in this study can assist policymakers in allocating resources more efficiently by identifying geographic areas where disease burdens overlap, thereby enabling coordinated responses across multiple NCD categories. These findings underscore the value of incorporating spatial epidemiological evidence into regional health planning frameworks to support more targeted, data-driven strategies for NCD prevention and control.

## 5 Conclusion and recommendations

This study demonstrates the value of a multivariate Bayesian spatio-temporal approach for understanding how place structures the burden of non-communicable diseases in East Africa. By modeling cardiovascular diseases, cancers, chronic respiratory diseases, and diabetes jointly over two decades, we provide a nuanced account of both shared and disease-specific mortality risks and how these patterns have shifted over time. The results show pronounced geographic inequalities in NCD mortality that reflect the combined influence of environmental conditions, urbanization trajectories, and socioeconomic context. Notably, persistent residual spatial effects in settings such as Rwanda and Uganda after adjustment for measured covariates point to unobserved structural, ecological, or health-system drivers that warrant further investigation.

Joint modeling reveals that NCD risks co-vary across places in ways that are not captured by single-disease analyses, while also highlighting meaningful divergences between disease groups. These findings support a set of place-sensitive recommendations. First, NCD strategies should be geographically targeted, prioritizing persistently high-risk clusters and recognizing that the determinants of risk differ across disease categories. Second, policy responses should be cross-sectoral, linking health interventions with environmental management, urban planning, and social protection where contextual risks are concentrated. Third, strengthening routine surveillance and investing in spatially referenced data systems can improve the ability to monitor inequalities and evaluate interventions over time.

As East Africa continues its epidemiological and urban transitions, effective action will depend on integrating spatial evidence, environmental indicators, and health-systems metrics to deliver equitable, context-specific responses. The modeling framework presented here is scalable and interpretable, and can support routine, place-based decision-making for NCD prevention and health-system planning across the region.

## Data Availability

The original contributions presented in the study are included in the article/supplementary material, further inquiries can be directed to the corresponding author.
